# Editorial: Progress in molecular mechanisms and targeted therapies for solid tumor microenvironments

**DOI:** 10.3389/fcell.2026.1932659

**Published:** 2026-07-27

**Authors:** Limin Hu, Kairan Li, Tomoaki Mitsunaka, Shuiyu Xu, Ren Zhao, Haosheng Li

**Affiliations:** 1 Department of General Surgery, Ruijin Hospital, Shanghai Jiao Tong University School of Medicine, Shanghai, China; 2 Shanghai Institute of Digestive Surgery, Ruijin Hospital, Shanghai Jiao Tong University School of Medicine, Shanghai, China

**Keywords:** biomarkers, precision oncology, single-cell sequencing, targeted therapy, tumor microenvironment (TME)

## Introduction

Solid tumors are not merely collections of malignant cells. They are dynamic ecosystems comprising cancer cells, stromal cells and immune cells, vascular compartments, extracellular matrix components, metabolites and soluble mediators. The tumor microenvironment (TME) actively shapes tumor initiation, progression, metastasis, immune evasion and therapeutic resistance. Targeted therapies have transformed the treatment of many solid tumors, yet durable clinical benefit is often constrained by tumor heterogeneity, adaptive resistance and microenvironment-mediated protection. Understanding how tumor-intrinsic alterations interact with stromal, immune, metabolic and stress-response programs is therefore essential for developing more precise and effective treatments.

The Research Topic “*Progress in Molecular Mechanisms and Targeted Therapies for Solid Tumor Microenvironments*” brings together 15 original studies, reviews and systematic analyses. Collectively, the Research Topic covers molecular target discovery, immune and stromal regulation, extracellular matrix remodeling, cellular stress adaptation and biomarker development. They also examine machine-learning approaches to patient stratification and translational platforms for precision oncology. Together, these contributions reflect a broader shift in solid tumor therapy, from targeting isolated oncogenic drivers to therapeutically engaging the tumor ecosystem. Unlike earlier syntheses that often focused on a single pathway or cell type, this Research Topic spans tumor-intrinsic signaling, stromal–immune crosstalk, stress adaptation, biomarker development and translational modeling. It therefore provides an integrated view of how solid-tumor therapy is shifting from the targeting of individual oncogenic drivers toward therapeutic modulation of the tumor ecosystem. The following sections trace this continuum from cell-autonomous vulnerabilities, through matrix and immune regulation, to biomarker-guided stratification and clinically relevant platforms for TME-directed precision therapy.

## Tumor-intrinsic signaling pathways and targeted vulnerabilities

A central theme of this Research Topic is the identification of molecular dependencies that sustain tumor growth and create therapeutic opportunities. Destefani et al. investigated Frizzled-4 (FZD4), a Wnt receptor with context-dependent functions in epithelial cancers, in oral squamous cell carcinoma. Pharmacological inhibition of FZD4 delayed cell-cycle progression and reduced proliferative capacity. These effects were associated with altered retinoic acid metabolism, implicating the Wnt–retinoic acid axis as a potential regulatory vulnerability. The study illustrates how tumor-intrinsic signaling can generate downstream metabolic dependencies with therapeutic relevance (Destefani et al.). In prostate cancer, Wu et al. reviewed the molecular rationale and translational potential of several targeted strategies. These included androgen receptor inhibition, PI3K–AKT–mTOR pathway modulation, DNA damage repair targeting and prostate-specific membrane antigen-directed therapy. The authors also considered proteolysis-targeting chimeras (PROTACs) and immunotherapy combinations. Their synthesis indicates that progress in precision treatment will increasingly depend on molecular stratification, longitudinal monitoring of resistance and rational combination therapy (Wu et al.). The clinical trade-offs of combination treatment were examined by Peng et al. in a systematic review and meta-analysis of advanced EGFR-mutant non-small-cell lung cancer. Across 10 randomized controlled trials, first-generation EGFR tyrosine kinase inhibitors plus chemotherapy yielded progression-free and overall survival comparable to those achieved with third-generation EGFR inhibitors. The combination produced a higher objective response rate but also more grade 3 or higher treatment-related adverse events. These findings highlight the need to weigh efficacy against toxicity, accessibility and patient preference when selecting targeted therapies (Peng et al.).

## Stromal, immune, and extracellular matrix regulation in therapeutic resistance

Several contributions extend beyond tumor-cell-intrinsic alterations to examine how the TME shapes treatment response. Liu and Yu reviewed the role of tumor-associated macrophages (TAMs), a dominant immune population in many solid tumors, in glioma chemoresistance. M2-like TAMs can alter drug metabolism and clearance, secrete cytokines and growth factors, maintain glioma stemness, facilitate adaptation to hypoxia and remodel the extracellular matrix. Strategies that modulate TAM recruitment, polarization, abundance, metabolism or phagocytic activity may therefore help to overcome resistance. Their clinical value, however, will depend on selectively disrupting tumor-promoting functions while preserving protective immunity (Liu and Yu). The extracellular matrix is likewise an active determinant of tumor progression and treatment efficacy. Wang et al. reviewed tenascin-C (TNC), an extracellular matrix glycoprotein widely expressed in tumor stroma. TNC contributes to mechanical remodeling, immune suppression, metabolic adaptation and radiation response within the TME. Its effects on matrix stiffness, immune-cell recruitment, hypoxic signaling, glucose metabolism, pH regulation and radiosensitivity challenge the view of the matrix as a passive scaffold. Instead, matrix-derived signals may constitute therapeutically actionable components of the tumor ecosystem (Wang et al.). Cellular stress responses provide another route to microenvironmental adaptation. Zhang et al. reviewed endoplasmic reticulum (ER) stress in lung cancer. Hypoxia, oxidative stress, nutrient deprivation, acidosis, oncogenic signaling and anticancer treatment can activate the unfolded protein response and ER-associated degradation. Depending on its intensity and duration, ER stress can promote autophagy, cellular reprogramming and adaptation, or trigger cell death. These context-dependent outcomes influence epithelial-to-mesenchymal transition, metabolism, immune regulation and drug resistance, complicating therapeutic targeting of this pathway (Zhang et al.). Wu et al. examined the interplay between redox signaling and cellular senescence in drug resistance. Therapy-induced senescent cells can acquire a senescence-associated secretory phenotype and release cytokines, chemokines, growth factors and matrix-remodeling enzymes. This secretory program can reshape the TME and facilitate immune evasion. The resulting feedback between redox imbalance and senescence suggests a possible therapeutic strategy. Co-targeting malignant cells and senescent stromal populations may help to limit relapse, although the timing and selectivity of such interventions require careful evaluation (Wu et al.).

## Non-coding RNAs, inflammatory mediators, and diagnostic biomarkers

Biomarkers that capture the immune and inflammatory state of the TME could inform diagnosis, prognosis and treatment selection. Chen et al. reviewed the roles of microRNA-155 (miR-155) in cervical cancer, including its involvement in apoptosis, migration, invasion, drug resistance and immune regulation. These diverse functions support its potential use as both a biomarker and a therapeutic target. However, the effects of miR-155 vary across tumor types and disease stages. This context dependence illustrates a broader challenge in translating non-coding RNA biology into clinically robust interventions (Chen et al.). In a complementary diagnostic study, Chen et al. identified calcitonin receptor-like receptor (CALCRL) as an immune- and inflammation-related marker for distinguishing uterine leiomyosarcoma from uterine leiomyoma. The authors integrated differential expression analysis, immune- and inflammation-related gene sets, machine-learning algorithms, validation cohorts, immunohistochemistry and immune-infiltration analysis. Their findings suggest that CALCRL expression may help to distinguish malignant from benign uterine smooth-muscle tumors. More broadly, the study shows how immune and inflammatory features can be integrated into diagnostic biomarker development (Chen et al.).

## Single-cell sequencing and machine learning reveal hidden TME heterogeneity

Advances in single-cell profiling and machine learning are revealing forms of TME heterogeneity that are obscured by bulk measurements. Wang et al. generated a single-cell RNA-sequencing atlas of tracheal squamous cell carcinoma from 70,682 high-quality cells across malignant and non-malignant samples. The analysis defined major cell populations and transcriptional regulators associated with chemotherapy-induced transitions in T-cell states. It also identified intercellular communication patterns specific to malignant samples. This atlas provides a molecular framework for investigating a rare and understudied tumor type (Wang et al.). Other studies used machine learning to derive immune-related prognostic or predictive signatures. Hong et al. integrated single-cell and bulk transcriptomic data with multiple machine-learning methods to construct a prognostic signature for clear-cell renal cell carcinoma. The model stratified prognosis, predicted immunotherapy response and indicated potential drug sensitivities. Single-cell analysis localized the signature predominantly to endothelial cells and highlighted DLL4–Notch and JAG–Notch signaling as prominent ligand–receptor interactions (Hong et al.). In stomach adenocarcinoma, Xue et al. developed a machine-learning-derived immune-evasion signature. A high immune-evasion score was associated with poor survival, reduced infiltration of CD8-positive T cells and dendritic cells, increased M2 macrophage abundance and an immunosuppressive TME. By contrast, low-score tumors had a higher tumor mutational burden and lower Tumor Immune Dysfunction and Exclusion (TIDE) scores, features associated with a more favorable immunotherapy response (Xue et al.). Ding et al. similarly constructed an immune-escape-related signature for lung adenocarcinoma. Low-risk tumors showed greater natural killer-cell and CD8-positive T-cell infiltration, stronger immune activation and a higher tumor mutational burden. They were also predicted to respond more favorably to immunotherapy. Functional experiments further implicated poliovirus receptor-related 1 (PVRL1) in tumor-cell proliferation and programmed death-ligand 1 (PD-L1) regulation (Ding et al.). Collectively, these studies suggest that immune-state signatures may complement conventional genomic biomarkers. Prospective validation and harmonization across platforms will be essential before such models can guide clinical decisions.

## Translational models and delivery systems for precision therapy

Mechanistic insights must ultimately be tested in clinically relevant models and connected to effective delivery strategies. Singh et al. reviewed precision-oncology approaches based on patient-derived organoids and functional biomaterials. Conventional two-dimensional cultures and animal models often incompletely reproduce patient-specific heterogeneity, immune context and tumor–stroma interactions. Integrating patient-derived organoids with extracellular matrix mimetics, biomaterials, co-culture systems, microfluidics and organ-on-chip platforms could improve drug-response profiling and biomarker discovery. Such systems may eventually support clinical decision-making, provided that they become sufficiently reproducible, scalable and representative (Singh et al.). Wu et al. reviewed plant-derived extracellular vesicles as emerging tumor-targeted delivery systems. Their natural origin, biocompatibility, bioactive cargo and capacity to modulate tumor cells or TME homeostasis make them attractive candidates for cancer therapy. However, their biodistribution, targeting specificity, cargo standardization and clinical manufacturability remain unresolved. Addressing these issues will determine whether plant-derived extracellular vesicles can become practical platforms for drug delivery and TME remodeling (Wu et al.).

## Summary and prospective

The contributions to this Research Topic show that solid tumor therapy is moving toward an ecosystem-oriented framework. Tumor progression and resistance arise not only from oncogenic mutations or individual signaling pathways, but also from interactions among malignant, immune, stromal and vascular compartments. Extracellular matrix architecture, metabolic niches, stress adaptation and immune-evasion programs further shape these interactions. The studies collected here identify potential molecular targets and illustrate emerging approaches to TME remodeling, biomarker discovery and precision treatment.

Four priorities follow from this body of work. First, mechanistic studies should connect tumor-intrinsic signaling to immune, stromal, metabolic and stress-response programs rather than examining these processes in isolation. Second, biomarker development should progress from single-gene markers to multidimensional signatures incorporating immune infiltration, inflammation, spatial organization, intercellular communication and treatment-induced adaptation. Third, patient-derived organoids, organ-on-chip systems, single-cell and spatial omics, and machine learning should be integrated more closely into preclinical and clinical workflows. Finally, biomarker-guided combination therapies will be needed to improve efficacy while limiting toxicity. Achieving these goals will require prospective validation, standardized analytical pipelines and models that preserve the spatial and temporal complexity of the human TME.

We thank all authors, reviewers, editors and members of the editorial team for their valuable contributions to this Research Topic. Continued investigation of molecular mechanisms and targeted therapies in solid tumor microenvironments may reveal new ways to overcome resistance and refine patient stratification. These advances could ultimately support more effective and durable treatments for patients with solid tumors.

